# Interprosthetic humeral fracture revision using a tibial allograft total elbow prosthetic composite in a patient with hemophilia A : a case report

**DOI:** 10.1186/1752-1947-6-319

**Published:** 2012-09-25

**Authors:** Justin LeBlanc, Shannon Puloski, Kevin Hildebrand

**Affiliations:** 1 Department of Surgery, Section of Orthopedic Surgery, Health Sciences Centre, 3330 Hospital Drive NW, Calgary, AB, T2N 4N1, Canada; 2 Department of Surgery, Section of Orthopedic Surgery, 3280 Hospital Drive NW, Calgary, AB, T2N 4Z6, Canada

## Abstract

**Introduction:**

Interprosthetic fractures of the humerus are rare. Revisions of total elbow arthroplasty components in these cases are difficult. We report the first case of a patient with hemophilia who underwent a revision with a tibial allograft prosthetic composite without the need for hardware augmentation.

**Case presentation:**

A 43-year-old Caucasian man with a history of hemophilia and transfusion-related human immunodeficiency virus and hepatitis B and C presented with an interprosthetic fracture of his humerus after months of pain between his total elbow and total shoulder arthroplasties. Because of the poor remaining bone stock available in his distal humerus, a revision using a barrel-staved tibial allograft prosthetic composite was performed. Our patients’ factor VIII level was optimized before the operation and he suffered no major long-term complications at 28 months. His only complication was an incomplete radial nerve palsy that ultimately recovered and left him with some numbness on the dorsum of his hand.

**Conclusion:**

Careful use of an allograft prosthetic composite is a very reasonable option when a patient experiences an interprosthetic fracture. We have successfully performed revision total elbow arthroplasty for a patient with hemophilia with an interprosthetic fracture using a tibial allograft and no additional fixation, which resulted in his return to full activities of daily living, minimal pain and full incorporation of the allograft to host bone.

## Introduction

Arthroplasty is common in patients with hemophilia, secondary to repeat bleeding into joints that causes joint arthropathy. Severe arthropathy leads to pain, loss of range of motion, decreased strength and eventual disability. The joints most commonly affected are the knee, elbow and ankle, followed by the shoulder [1,2]. Total elbow arthroplasty (TEA) for patients with hemophilia results in better pain control, and an increased range of motion and function [2]. Complications are common after TEA for patients with hemophilia, including nerve palsies, deep vein thrombosis, infection, chronic pain, aseptic loosening, and periprosthetic fractures [1-3].

Interprosthetic fractures of the upper extremity are very rare. No incidence has been reported, but a few case series have described this fracture and techniques of revision [4-6]. Risk of periprosthetic total shoulder arthroplasty (TSA) fractures range from 0.6% to 2.4%, while periprosthetic risk of TEA is 0.65% for the humeral components [7,8]. As with the lower extremity, periprosthetic fractures need to be examined for location of the fracture, remaining bone stock and for component loosening [9]. Revisions of TEA have been adequately performed using hardware [4], strut allograft alone [10], and strut allograft with implant revision [9]. When there is massive bone loss, other modalities must be considered. Total humeral endoprosthesis [11] and allograft prosthetic composites (APCs) have been used [5,12,13]. Unfortunately, complications have frequently been reported through these case series. These include nerve palsies, triceps failure, allograft failure, infection, non-union, loosening, inability to return to preoperative activity level, and olecranon bursitis [7,9,12,13].

We present a case of an interprosthetic fracture of the humerus in a patient with hemophilia A (factor VIII deficiency), revised using a tibial APC without hardware, allowing the use of standard TEA components. Our patient went on to have functional range of motion, minimal pain, no infections, and complete incorporation of the allograft into the native humerus.

## Case presentation

**Figure 1:**
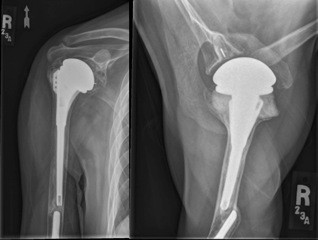
Anteroposterior and axillary radiographs when the patient first presented with some pain in the humerus.

Our patient was a 43-year-old Caucasian man with severe hemophilia A and transfusion-related hepatitis B, hepatitis C and human immunodeficiency virus (HIV). His HIV viral load was undetectable and cluster of differentiation 4 count was above normal at the time of surgery. No complications due to the hepatitis were noted at the time of surgery. He has longstanding hemophilia and has had multiple joints replaced over the years. On his right arm, he has received both a cemented TEA, seven years prior to presentation (Conrad-Morrey; Zimmer, Warsaw, IN), and a TSA 12 years prior to presentation. He was seen in the spring one year before presentation with arm pain of approximately one year’s duration and diagnosed with a pending interprosthetic fracture (Figure 1).

**Figure 2:**
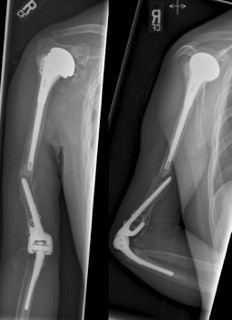
Patient presents with an interprosthetic fracture.

Within seven months, our patient suffered an interprosthetic fracture after a fall (Figure 2). He was seen on an urgent basis and a discussion about revision arthroplasty was started. He was initially treated nonoperatively, using a Sarmiento brace for comfort while awaiting surgery. Before the operation, he had a normal neurological examination in his right hand, known previous triceps weakness, and there were no signs of humeral TSA component loosening. The hemophilia clinic was consulted for presurgical optimization. With their management, it was decided to go forward with the surgery. After organizing the hemophilia clinic and internal medicine preoperative consultations, surgery eventually consisted of an APC reconstruction of his right distal humerus using a tibial allograft.

Our patient received factor VIII the evening before and the morning of the surgery. His factor VIII level was 2.34U/mL (normal range, 0.54U/mL to 1.47U/mL) on the morning of surgery. He received preoperative antibiotics for Gram-positive bacteria, and was then placed in the lateral decubitus position with an arm bolster. The previous posterior skin incision was used and the ulnar and radial nerves were isolated and protected. A triceps-sparing approach was used to expose the joint, working on the medial and lateral sides of his humerus and leaving the insertion intact on the olecranon. The axis pin was dissociated and the humeral component was loose. The cement mantle came out with little difficulty. The ulnar component was solid with no loosening, so it was left in place. The existing distal humerus stalk was then inspected; we noted a large posterior defect and thin cortical shell both medially and laterally, with very poor bone quality anterior in the distal segment. The previous fracture was found to be united in malposition.

Given the malposition and poor quality bone, we elected to proceed with an APC. The residual humeral bone was osteotomized to remove the malunion and cortical shell. Intraoperative tissue samples were sent to pathology and no evidence of acute infection was identified. This left a stable straight cylinder of bone about 1cm to 2cm distal to the shoulder prosthesis. The allograft for the distal humerus was fashioned from an intact tibia diaphysis. All fibrous tissue was removed from the allograft followed by preparation of the canal. A high-speed burr was used to prepare the proximal extent of the allograft to barrel-stave the allograft over the humerus itself. The distal tibial allograft segment was then osteotomized to leave the appropriate length of the humerus distally. A small size humeral component was used to match the ulnar component. The tibia allograft was prepared using the appropriate broaches. Rotation of the tibia allograft was confirmed and the trial component was inserted, followed by reduction of the elbow prosthesis. Near full extension was achieved and flexion beyond 130°. After cleaning the canal, the allograft was retrograde filled with commercially available cement containing gentamicin. The allograft was barrel-staved onto his humerus first. The cement was then placed into the allograft followed by positioning of the implant into the allograft. A small size distal humerus component was then cemented into place. Care was taken to ensure no extravasation of cement at the host-graft and around his radial nerve. The nerve rested free on the proximal aspect of the allograft at the end of the procedure. His ulnar nerve was transposed anteriorly and ensured to be free from tension with a range of motion of his elbow. The deep medial and lateral fascia was closed with a #1 Vicryl suture; a Hemovac drain was placed deep and superficial to his triceps fascia; the subcutaneous tissue was closed with 2-0 Vicryl suture and his skin closed with a 4-0 Monocryl suture.

Our patient was brought to the postoperative recovery room in stable condition. Unfortunately, an immediate postoperative radial nerve palsy was noted. We placed our patient into a wrist drop splint and he followed up at six weeks with minimal pain and no signs of infection. The postoperative radial nerve palsy persisted, with a normal median and ulnar nerve examination. The wrist drop splint was maintained and electromyogram and nerve conduction studies were organized. Three months after the operation, our patient noted subjectively that his wrist extension and dorsal numbness was improving so he was not using his splint as much. At the time of his electromyogram and nerve conduction studies four months after the surgery, he had grade 2/5 to 3/5 bilateral triceps strength (known prior to previous surgery), grade 2/5 wrist extension, grade 0/5 extensor digitorum communis, extensor indicis proprius and thumb extensors, with decreased pin-prick sensation to his superficial radial and posterior cutaneous forearm. Sensory studies resulted in normal findings for his median and ulnar nerves for both his arms. His right radial nerve demonstrated decreased amplitude at 8μV (left radial nerve, 40μV). Results from bilateral median and ulnar nerve motor studies were normal. Right radial nerve motor studies showed an absent response, fitting with severe radial neuropathy, which is axonal in nature. Some activity of the brachioradialis and posterior cutaneous nerve of his forearm placed the injury between the spiral groove and takeoff of the brachioradialis. Some denervation was demonstrated in his extensor indices proprius. Our impression was that the palsy was either due to a traction injury during the revision or due to the hemophilia, with hemorrhage within the nerve itself secondary to traction.

**Figure 3:**
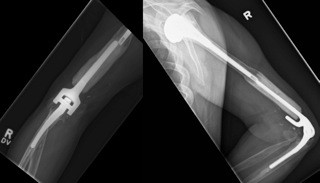
Anteroposterior and lateral radiographs of the elbow at 28 and 24 months post operation, demonstrating incorporation of the allograft into the host humerus.

At 28-months follow-up, our patient stated he was able to perform all activities of daily living (ADL) without restrictions and had not had any concerns with infection since the surgery. His pain was well controlled and he denied any symptoms of instability of his elbow or weakness in his hand. He had near complete resolution of his radial nerve palsy. He had no concerns with his elbow and was quite satisfied. On examination, his incision had healed well, with no signs of current infection, and he had a regular radial pulse. His wrist examination demonstrated slight decreased sensation to the first dorsal web space, and normal median and ulnar sensation. His motor examination demonstrated 5/5 wrist extension, 4/5 extensor pollicis longus, and 5/5 finger extension with no lag. The remaining wrist and hand examination were normal. His wrist extension was 90°, flexion 90°, pronation 95°, and supination 0°. An elbow examination demonstrated 4/5 flexion and 2/5 extension (left 3/5). The arc of motion of his right elbow was 31° to 131° while the left was 15° to 141°. X-rays revealed complete incorporation of the APC to the native humerus with no evidence of loosening and resorption of the olecranon (Figure 3).

## Discussion

Interprosthetic fractures of the upper extremity are rare. To the best of our knowledge, no other articles have discussed the use of an APC using a tibial allograft to revise an interprosthetic fracture in a patient with hemophilia without the use of supplemental hardware. Our patient has a 28-month follow-up, with resolved radial nerve palsy with no other complications and excellent incorporation of the allograft, and states that he functions well doing all ADL without restriction.

With increasing numbers of prostheses being implanted in patients with hemophilia and rheumatoid arthritis, interprosthetic fractures will likely increase in incidence. Managing massive bone loss remains a difficult challenge. Having multiple modalities to choose from will ensure that the selection from these options will fit specific patients. The idea of using cadaveric bone for revising elbows had been around since 1925, and a few studies have examined using an APC for revision TEA [5,12,13]. Some studies have suggested cementing the humeral component first, then impacting the allograft onto the humerus [5]. In our case, we found it easier to obtain an appropriate length by securing the allograft to the host bone first. The fit to host bone is very important. Our patient received a metaphyseal size-matched allograft that was barrel-staved over his native humerus. The benefit of this was that no hardware was required and the overlap of the host-graft junction created greater surface area for ingrowth (Additional file 1: Illustration 1). Using a larger diameter graft (tibia or femur) allows us to impact the cortical bone of the humerus into the cancellous bone of the bulk graft. Other techniques have been utilized and described. Kieser *et al*. employed an invagination method, using a larger diameter humerus allograft to fit over the host humerus, then securing it with hardware [5].

Also important for the stability of the allograft/host bone interface is a proper step cut, as previously described in the lower extremities [12]. We must remember that regions of the upper extremity have more difficulties than the lower extremities, with smaller bones, more use of primary cemented implants, and more iatrogenic nerve injuries [9]. The radial nerve palsy in our patient was likely from a traction type injury. His condition of hemophilia could have added to the severity, with hemorrhage within the nerve itself. Fortunately, our patient went on to have near complete resolution of his palsy. Due to the proximity of his radial nerve to the graft, we were prevented from considering strut grafts to further support the APC-host junction.

## Conclusion

To the best of our knowledge, this is the first report of the use of a tibial APC without hardware for an interprosthetic humerus fracture. Careful use of the APC with gross bone loss is a very reasonable option when a patient has an interprosthetic fracture. We found that revision TEA in a patient with hemophilia with an interprosthetic fracture using a tibial allograft and no additional fixation resulted in a return to full ADL, minimal pain and full incorporation of the allograft to the host bone.

## Consent

Written informed consent was obtained from the patient for publication of this case report and accompanying images. A copy of the written consent is available for review by the Editor-in-Chief of this journal.

## Abbreviations

ADL: activities of daily living; APC: allograft prosthetic composites; HIV: human immunodeficiency syndrome; TEA: total elbow arthroplasty; TSA: total shoulder arthroplasty.

## Competing interests

The authors declare that they have no competing interests.

## Authors’ contributions

JL reviewed the available literature, and drafted the manuscript. SP and KH assisted with providing in-depth background knowledge of the patient and procedure as well as a critical review of the manuscript. All authors read and approved the final manuscript.

## Supplementary Material

Additional file 1 **Illustration 1.** Technique of barrel staving (courtesy of Dr. Ryan Martin). Click here for file
