# Supplementary figures and images for: Interprosthetic humeral fracture revision using a tibial allograft total elbow prosthetic composite in a patient with hemophilia A : a case report
Source: J Med Case Rep. 2012 Sep 25;6:319. doi: 10.1186/1752-1947-6-319 (PMC3492012; doi:10.1186/1752-1947-6-319)

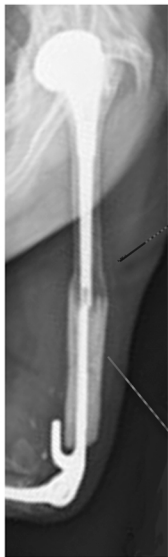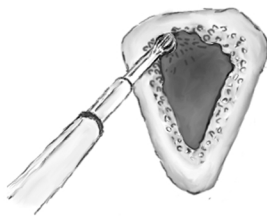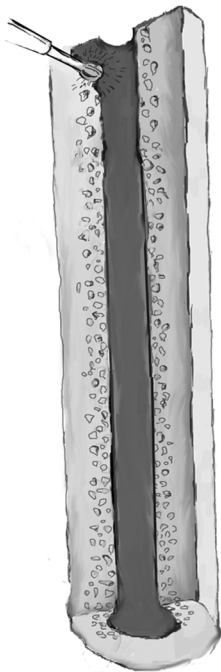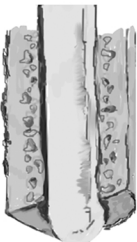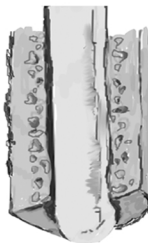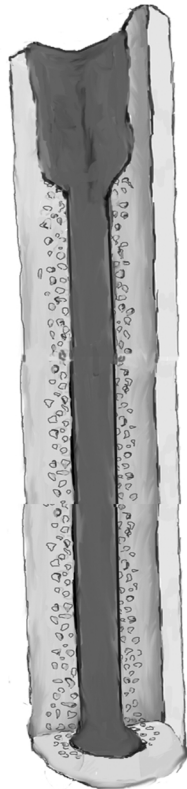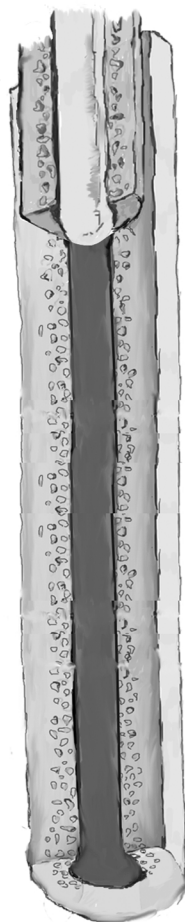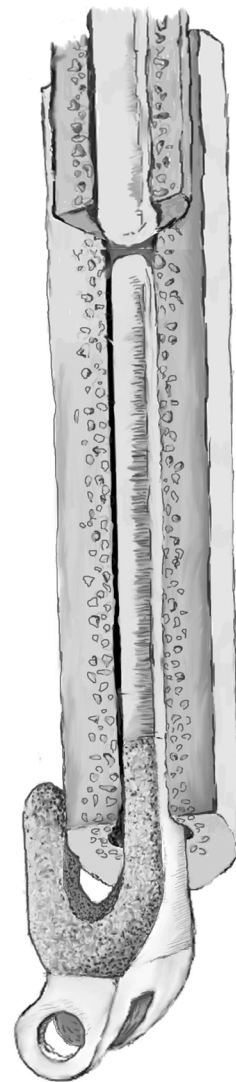

R. Martin

Supplement: Additional file 1 — Illustration 1. Technique of barrel staving (courtesy of Dr. Ryan Martin). [file 1752-1947-6-319-S1.pdf]
